# Devotion to Duty

**Published:** 1906-02

**Authors:** 


### Devotion to Duty.

"The longer on this earth we live
  And weigh the various qualities of men,
The more we feel the high, stern-featured beauty
  Of plain devotedness to duty,
Steadfast and still, nor paid with mortal praise,
  But finding amplest recompense
For life's ungarlanded expense
  In work done squarely and unwasted days."

*Good Health.*

